# STAT5A/5B-specific expansion and transformation of hematopoietic stem cells

**DOI:** 10.1038/bcj.2016.124

**Published:** 2017-01-06

**Authors:** S Ghanem, K Friedbichler, C Boudot, J Bourgeais, V Gouilleux-Gruart, A Régnier, O Herault, R Moriggl, F Gouilleux

**Affiliations:** 1 UCBG, R&D Unicancer, Paris, France; 2 University of Veterinary Medicine, Medical University of Vienna and Ludwig Boltzmann Institute for Cancer Research, Vienna, Austria; 3 INSERM UMR 1088, Université de Picardie J. Verne, Amiens, France; 4 CNRS UMR 7292 GICC, Université François Rabelais, Tours, France; 5 CHRU de Tours, Service d'Hématologie Biologique, Tours, France; 6 CHRU de Tours, Laboratoire d'Immunologie, Tours, France; 7 EA 4666, Université de Picardie J. Verne, Amiens France

Signal transducer and activator of transcription 5A and 5B (STAT5A/5B) are encoded by closely related and chromosomally juxtaposed genes. Both proteins play a major role in normal hematopoiesis.^1^ STAT5A/B-deficient hematopoietic progenitors failed to repopulate the bone marrow in competitive repopulation assays and have a reduced ability to respond to early-acting cytokines such as interleukin (IL)-3 and stem cell factor (SCF), indicating that STAT5 proteins are important regulators of hematopoietic stem cell (HSC) biology.^2, 3, 4^ A myeloproliferative disorder was linked to HSC proliferation in regard to hematopoietic transformation and gain-of-function studies with a STAT5A mutant.^5, 6^ In humans, down-modulation of STAT5 expression impairs long-term self-renewal and maintenance of cord blood-derived CD34^+^ cells.^7, 8^ Moreover, changes in the expression levels of STAT5 proteins differently affect self-renewal, proliferation and lineage commitment of human HSCs.^9^ Although STAT5A and STAT5B have overlapping functions during hematopoiesis, the respective contribution of each molecule in HSC biology remains vague. Thus, we analyzed the capacity of STAT5A and STAT5B to control expansion of human hematopoietic stem/progenitor cells (HS/Pc) and transformation of murine HSC using oncogenic STAT5A/5B variants.

To address how STAT5A and STAT5B differentially affect human HS/Pc proliferation and/or survival, we analyzed the effects of transduced TAT-STAT5A/5B recombinant proteins in cord blood-derived CD34^+^ cells cultured with SCF. We first verified that STAT5A/5B proteins were endogenously expressed and activated by SCF in CD34^+^ cells (Supplementary Figures S1A and B). We also observed that SCF alone had a weak capacity to support the growth of CD34^+^ cells *in vitro* and this was accompanied in long-term culture by the graduate downregulation of endogenous STAT5A/5B expression (Supplementary Figure S1C). In sharp contrast, transduction of a recombinant TAT-STAT5A protein induced a strong expansion of CD34^+^ cells cultured with SCF^10^ (Supplementary Figure S1G). This effect requires tyrosine phosphorylation of STAT5A because transduction of a recombinant TAT-STAT5A protein mutated on the critical tyrosine activation residue 694 (TAT-STAT5A^Y694F^) failed to induce expansion of CD34^+^ cells (Supplementary Figures S1D–G). Moreover, CD34^+^ cells transduced with TAT-STAT5A protein were not able to grow in the absence of SCF (data not shown). These data indicated that sustained expression and activation of STAT5A are sufficient to promote CD34^+^ cell growth. We then asked whether TAT-STAT5A or TAT-STAT5B recombinant proteins maintain similar impact on CD34^+^ cells. A schematic overview on the modular design of TAT-STAT5A/5B recombinant proteins used in this study is shown in Supplementary Figure S2A. Both proteins were produced in bacteria, and purified as described.^10^ The purity and identity of both recombinant proteins were confirmed by Coomassie gel staining and western blot using either anti-HA or anti-STAT5-specific antibodies (Supplementary Figure S2B). The purified protein concentration was 10 nM throughout all experiments. Transduction efficiency in CD34^+^ cells was monitored by western blot using anti-HA and anti-STAT5 antibodies (Figure 1a). TAT-STAT5 proteins were detected 12 h post transduction and were present during 48 h.^10^ TAT-STAT5A and TAT-STAT5B proteins were then added to the culture medium containing SCF every 2 days to maintain expression of the recombinant proteins in CD34^+^ cells (Figure 1b). The extent of cell proliferation kinetics was determined at 20 days. A growth advantage was already observed after 10 days of culture when CD34^+^ cells were transduced with TAT-STAT5A (eightfold; Figure 1c). In contrast, the effect of TAT-STAT5B protein was almost negligible when compared to non-transduced cells at the same time point. Interestingly, we observed a significant increase in the number of CD34^+^ cells transduced with TAT-STAT5B protein at day 15, reaching an eightfold expansion at day 20. As control, transduced CD34^+^ cells were also cultured with the ligand of FLT3 receptor (FLT3-L) that does not activate STAT5 in CD34^+^ cells (Supplementary Figures S1B and D). Surprisingly, the results showed that both TAT-STAT5 proteins were able to induce a moderate expansion of CD34^+^ in the presence of this ligand. However, no significant differences were observed between both recombinant proteins. We concluded from these data that STAT5A and STAT5B have distinct effects on HS/Pc expansion. We next addressed whether transformation of HS/Pc and induction of leukemia in mice might be different by these two proteins. Murine HSC (Lin^−^ Sca^+^ Kit^+^ (LSK)) cells were infected with recombinant retrovirus expressing constitutively active STAT5A (cS5a) or STAT5B (cS5b) followed by IRES-GFP or GFP alone as control. GFP^+^ cells were sorted and cultured with SCF at indicated times. Uninfected LSK cells were cultured in the presence of SCF and IL-3 (SCM medium) as positive control (Figure 1e). SCF alone was able to stimulate the proliferation of LSK cells expressing cS5a or cS5b, but not the growth of control GFP cells (Figure 1e). Interestingly, after 10 days of cell cultures, LSK cells expressing cS5b stopped dividing, while the proliferation of cS5a-expressing cells was maintained. Transplantation of transduced LSK cells also demonstrated that both constitutively active STAT5 isoforms had distinct capacities to induce leukemia (Figure 1f). Overall, these data suggested that STAT5 isoforms have intrinsically distinct cell-growth-promoting properties that differentially affect HS/Pc biological activity.

STAT5A and STAT5B differ mainly at the carboxyl terminal transactivation domain (TAD).^11^ We then addressed whether the TAD of STAT5A might explain these functional differences. We generated different TAT-STAT5A mutants that were progressively deleted in the COOH-terminal region (Supplementary Figure S2A). Recombinant proteins were produced and purified. The purity and identity of each protein was verified on Coomassie blue-stained SDS-PAGE and western blot (Supplementary Figure S2C). In order to delineate precisely the COOH-terminal region involved in TAT-STAT5A-induced cell growth, CD34^+^ cells were transduced with TAT-STAT5A and deletion mutants (Figure 2a). TAT-STAT5AΔ749, TAT-STAT5AΔ768 and TAT-STAT5AΔ775 recombinant proteins were ineffective in inducing CD34^+^ cell growth (Figure 2b). In contrast, transduction of TAT-STAT5AΔ785 was almost as efficient as TAT-STAT5A in inducing expansion of CD34^+^ cells. These data suggested that the sequence between amino acid (aa) 775 and aa 785 is of critical importance for STAT5A-induced CD34^+^ cell growth. Importantly, deletion of this sequence also abrogated the leukemogenic potential of the hyperactive cS5a variant (Supplementary Figure S3). Sequence alignment displays a unique serine at position 780 and 779 in human and mouse STAT5A, respectively. This residue is absent in human and murine STAT5B (Figure 2c). Constitutive phosphorylation on Ser779/780 has been described in various leukemic cells and mutation of this residue abrogates the transforming potential of STAT5A.^12^ We also demonstrated that STAT5A is constitutively phosphorylated on Ser779/780 in normal human CD34^+^ cells (Figure 2d). The P21-activated kinase family members, PAK1 and PAK2, were previously identified as protein kinases responsible for phosphorylation on Ser779/780.^13, 14^ We found that inhibition of PAK1/2 activities did not affect this phosphorylation in CD34^+^ cells, indicating that Ser779/780 is under the control of distinct unidentified serine kinase(s) in normal human HS/Pc (Supplementary Figure S4A). We addressed whether phosphorylation of Ser779/780 is required for STAT5A-induced human CD34^+^ cell growth. Mutation of this serine residue was introduced in TAT-STAT5A (TAT-STAT5A^S779A^, Supplementary Figure S2A). The purity and identity of TAT-STAT5A^S779A^ recombinant protein were assessed by Coomassie gel staining and western blot (Supplementary Figure S2D). Transduction efficiency of TAT-STAT5A and TAT-STAssT5A^S779A^ proteins was monitored by western blot as shown in Figure 2e. Growth of CD34^+^ cells transduced with TAT-STAT5A or TAT-STAT5A^S779A^ was then evaluated in a time-course experiment (Figure 2f). Mutation of Ser779 abrogated the growth-promoting effect of TAT-STAT5A in CD34^+^ cells. Similarly, the TAT-STAT5AΔ785 recombinant protein with a Ser^779^ mutation (TAT-STAT5AΔ785^S779A^) failed to induce CD34^+^ cell proliferation (Supplementary Figures S4B and C).

In conclusion, our findings indicate that specific phosphorylation of STAT5A proteins at Ser^779^ differentially fine-tunes proliferation and transformation of HS/Pc with higher capacity over STAT5B. The highly related STAT5A/B proteins regulate hematopoiesis or neoplastic cell growth through distinct and specific signaling mechanisms, possibly explaining differences in leukemic disease development associated with gain-of-function STAT5A or STAT5B variants.

## Figures and Tables

**Figure 1:**
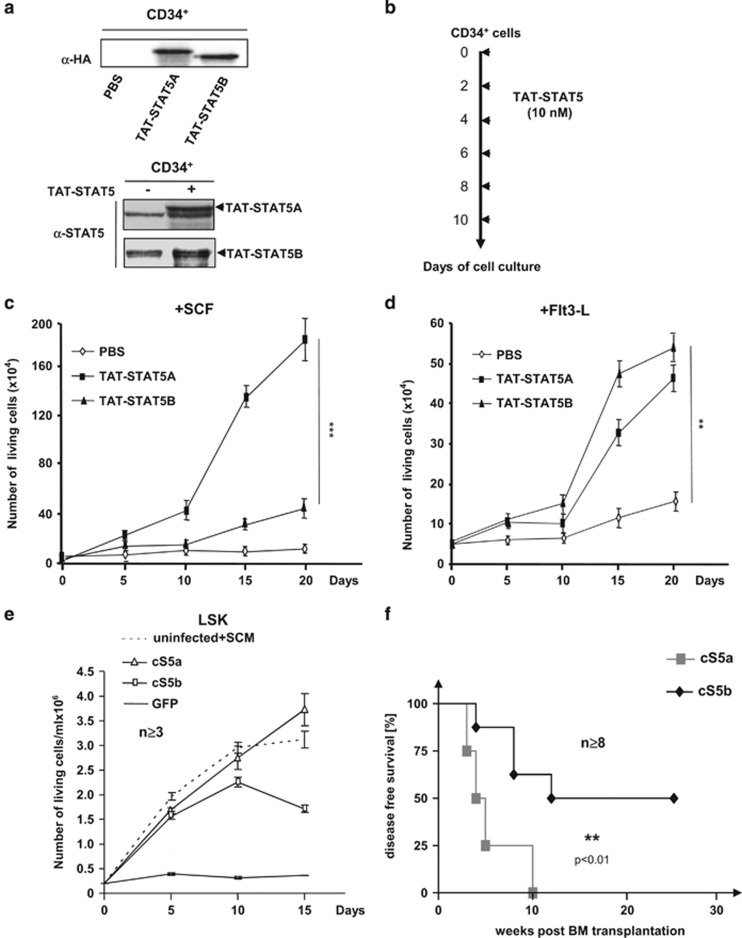
STAT5A and STAT5B have distinct effects on HS/Pc growth and transformation. (**a**) Extracts from human CD34^+^ cells transduced or not (phosphate-buffered saline) with recombinant TAT-STAT5A and TAT-STAT5B fusion proteins (10 nM) were analyzed by western blot with anti-HA (left) and anti-STAT5 antibodies (right). (**b**) Cell culture experimental design. TAT-STAT5 proteins (10 nM) were added to the culture medium every 2 days. (**c**, **d**) Transduced CD34^+^ cells were cultured in the presence of SCF (10 ng/ml) or Flt3-L (10 ng/ml) during 20 days. Cells were enumerated every 5 days (*n*=4, **P<0.01; ***P<0.001). (**e**) LSK cells expressing constitutively active STAT5A (cS5a) or STAT5B (cS5b) variants or GFP were grown in the presence of SCF alone. Control LSK cells were cultured with SCF and IL-3 (+SCM). Cell numbers were counted every 5 days (*n*=3). (**f**) Kaplan-Meier survival analysis of cS5a- vs cS5b-transplanted mice (*n*=8). All cS5a-transplanted mice succumbed to myeloproliferative disease within 10 weeks. Disease onset in cS5b-transplanted mice was significantly delayed with 50% latency (**P<0.01).

**Figure 2:**
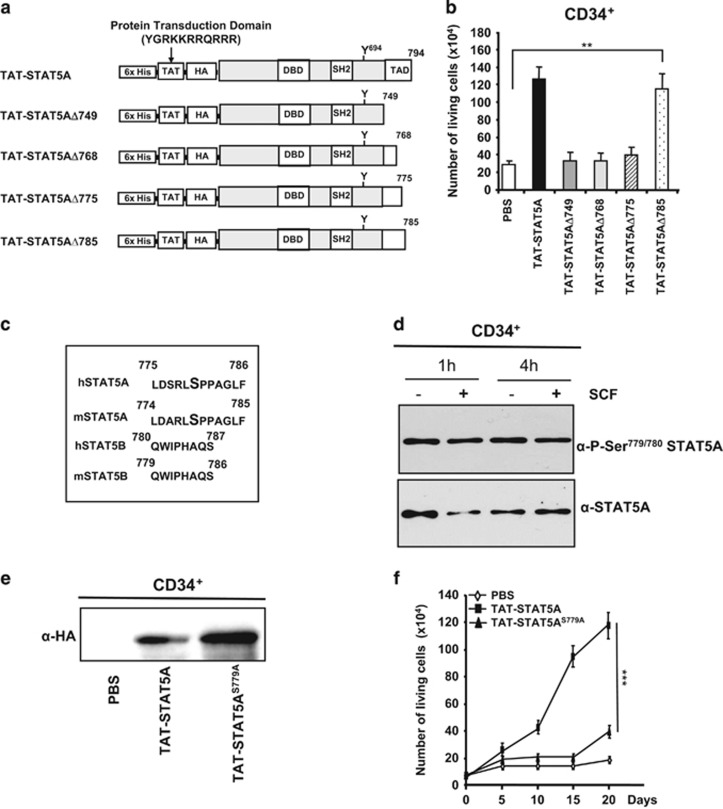
Phosphorylation of Ser779/780 is required for STAT5A-induced human HS/Pc growth. (**a**) Schematic representation of recombinant TAT-STAT5A protein and carboxyl-terminal deletion mutants. (**b**) Human CD34^+^ cells transduced with wild-type or mutant TAT-STAT5A proteins were cultured in presence of SCF for 10 days and the number of viable cells was counted (*n*=3, **P<0.01). (**c**) Sequence alignment of human and murine STAT5A/5B isoforms between aa 774 and aa 785 (STAT5A). (**d**) STAT5A is constitutively phosphorylated on Ser^780^ in human CD34^+^ cells. CD34^+^ cell extracts were analyzed by western blot with an anti-P-Ser^779780^-STAT5A antibody (*n*=3). (**e**) Human CD34^+^ cell extracts transduced or not (phosphate-buffered saline) with recombinant TAT-STAT5A and TAT-STAT5A^S779A^ proteins (10 nM) for 24 h were analyzed by western blot with an anti-HA antibody. (**f**) Growth kinetics of CD34^+^ cells transduced with recombinant TAT-STAT5A or TAT-STAT5A^S779A^ proteins (*n*=3; ***P<0.001). DBD, DNA binding domain; SH_2_, Src-Homology Domain 2; TAD, transactivation domain.
