# Supplementary material for: STAT5A/5B-specific expansion and transformation of hematopoietic stem cells
Source: Blood Cancer J. 2017 Jan 6;7(1):e514–. doi: 10.1038/bcj.2016.124 (PMC5301033; doi:10.1038/bcj.2016.124)
Supplement: Supplementary Information [file bcj2016124x1.doc]

**Supplementary information**

**STAT5A/5B-specific expansion and transformation of hematopoietic stem cells.**

**Materials and methods**

**Primary cell isolation and transplantation of hyperactive STAT5 variants**

Human CD34^+^ cells were isolated from umbilical cord blood samples as previously described ^1^. Human CD34^+^ cells were cultured in RPMI medium supplemented with 10% FCS, 2 mM L-glutamine, 10 i.u. penicillin, 10 µg/mL streptomycin, and 10 ng/mL rhSCF (Valbiotech). For transduction experiments, recombinant TAT-STAT5 proteins were added in culture medium every two days.

For murine retroviral infections, bone marrow (BM) cells were cultured in DMEM, 15% FCS containing IL-3 (R&D Systems, 25 ng/ml), IL-6 (R&D Systems, 50 ng/ml) and SCF (200 ng/ml). Transduction of BM cells and mice transplantations were performed as previously described ^2^. 48 h after retroviral infection, BM cells were analyzed for green fluorescent protein (GFP) expression by flow cytometry. LSK (Lineage^-^ Sca1^+^ c-Kit^+^) cells were then isolated from BM GFP^+^ cells by FACS before transplantations.

**Plasmids and purification of recombinant TAT-STAT5 fusion proteins**

The coding regions of STAT5A, STAT5B, STAT5A∆785, STAT5A∆775, STAT5A∆768, STAT5A∆749, STAT5A^Y694F^, STAT5A^S779A^ and STAT5A∆785^S779A^ were amplified by PCR and cloned into the pTAT-HA vector ^3^. TAT-STAT5 constructs were introduced in BL21(DE3)pLysS bacterias. Expression, purification of recombinant proteins and transduction in CD34^+^ cells were performed as described ^1,4^.

**Antibodies and Western blotting**

Cells were lysed in Laemmli’s buffer (0.0625 M, SDS (pH=6.8), 2%, 5% β-Mercaptoethanol, 10% glycerol) and analyzed by Western blot using the following antibodies: α-STAT5 (BD Biosciences), α-STAT5A, α-STAT5B (Zymed), α-HA tag (Roche), α-P-S^779/780^-STAT5A (Affinity Bio Reagent™), α-P-Y^694/699^-STAT5 and α-Actin (Cell Signaling)

**Supplementary Figure S1**

**A**) Human CD34^+^ cell extracts were analyzed by Western blot with anti-STAT5A and STAT5B antibodies. KU812 cell extracts served as a positive control and actin as a loading control.

**B**) Human CD34^+^ cells were stimulated with recombinant SCF (100 ng/ml), Flt3L (100 ng/ml) or IL-3 (10 ng/ml) for 30 min. Tyrosine phosphorylation of STAT5 was next evaluated by Western blot analysis with anti-P-Y^694/699^ STAT5A/5B antibodies. Membranes were reprobed with anti-STAT5 antibodies.

**C**) Human CD34^+^ cells were cultured in presence of SCF (10 ng/ml) at the indicated times. Extracts were then prepared and analyzed by Western blot with anti-STAT5 and anti-actin antibodies.

**D**) Schematic representation of recombinant TAT-STAT5A and tyrosine mutant TAT-STAT5A^Y694F^ proteins.

**E**) Purity of recombinant TAT-STAT5A, TAT-STAT5A^Y694F^ proteins was assessed by Coomassie blue staining of SDS-PAGE and identity of the proteins was confirmed by Western blot analysis using anti-HA and anti-STAT5 antibodies.

**F**) CD34^+^ cells were transduced or not (PBS) with TAT-STAT5A and TAT-STAT5A^Y694F^ proteins (10 nM) for 24 hours. The presence of recombinant TAT-STAT5 proteins in CD34^+^ cells was then determined by Western blot with indicated antibodies.

**G**) CD34^+^ cells cultured in the presence of SCF (10 ng/ml) were transduced or not (PBS) with TAT-STAT5A or TAT-STAT5A^Y694F^ (10 nM) proteins. Transduced cells were enumerated every 5 days (n=3,***p<0.001).

**Supplementary Figure S2**

**A**) Schematic representation of recombinant TAT-STAT5A, TAT-STAT5B and mutants TAT-STAT5A^S779A^ and TAT-STAT5AΔ785^S779A^ proteins. DBD: DNA Binding Domain; SH_2_: Src-Homology Domain 2; TAD: Transactivation Domain.

**B, C, D**) Purity of the recombinant TAT-STAT5 fusion proteins used in this study was assessed by Coomassie blue staining of SDS-PAGE. Purified TAT-STAT5A and TAT-STAT5B proteins had an expected molecular weight of 103 and 98 kDa, respectively. Identity of the proteins was verified by Western blot using anti-HA and anti-STAT5 antibodies.

**Supplementary Figure S3**

**A**) Kaplan-Meier Plot of cS5a- vs cS5a-Δ772-transplanted mice (n=4). All cS5a-transplanted mice died within 10 weeks. Disease onset in cS5a-Δ772–transplanted mice was significantly delayed (***p<0.0001)

**B**) WBC counts of transplanted mice at 8 weeks and 28 weeks after transplantation. PB hematology revealed a dramatic increase in WBCs in cS5a-transplanted mice after 8 weeks. By contrast, a slight increase in WBCs was observed in cS5a-Δ772–transplanted mice after 28 weeks only.

**Supplementary Figure S4**

**A**) Phosphorylation on Ser^780^ of STAT5A is independent of PAK1/2 activity in human CD34^+^ cells. CD34^+^ cells were incubated or not with the PAK inhibitor IPA3 (10 and 20 µM as indicated) during 1 or 2 hours in the presence or not of SCF. Cell lysates were prepared and analyzed by Western blot with indicated antibodies.

**B**) CD34^+^ cells were transduced or not (PBS) with TAT-STAT5A proteins, TAT-STAT5AΔ785 or TAT-STAT5AΔ785^S779A^ (10 nM) for 24 hrs. After extensive washes, the presence of recombinant proteins in CD34^+^ was detected by Western blot with anti-HA antibodies.

**C**) Growth of CD34^+^ cells transduced or not (PBS) with TAT-STAT5A, TAT-STAT5AΔ785 or TAT-STAT5AΔ785^S779A^ (10 nM) was determined in a time course experiment (n=3, ***p<0.001).

**Supplementary references**

1. Harir N, Boudot C, Friedbichler K, Sonneck K, Kondo R, Martin-Lannerée S *et al.* Oncogenic Kit controls neoplastic mast cell growth through a Stat5/PI3-kinase signaling cascade. *Blood* 2008; **112**: 2463–73.

2. Friedbichler K, Kerenyi MA, Kovacic B, Li G, Hoelbl A, Yahiaoui S, et al. Stat5a serine 725 and 779 phosphorylation is a prerequisite for hematopoietic transformation. *Blood* 2010; **116**:1548-58

3. Nagahara H, Vocero-Akbani AM, Snyder EL, Ho A, Latham DG, Lissy NA, et al. Transduction of full-length TAT fusion proteins into mammalian cells: TAT- p27Kip1 induces cell migration. *Nat Med.* 1998; **12**: 1449-52.

4. Harir N, Pecquet C, Kerenyi M, Sonneck K, Kovacic B, Nyga R *et al.* Constitutive activation of Stat5 promotes its cytoplasmic localization and association with PI3-kinase in myeloid leukemias. 2007; **109**: 1678–1686.
