# Supplementary figures and images for: STAT5A/5B-specific expansion and transformation of hematopoietic stem cells
Source: Blood Cancer J. 2017 Jan 6;7(1):e514–. doi: 10.1038/bcj.2016.124 (PMC5301033; doi:10.1038/bcj.2016.124)

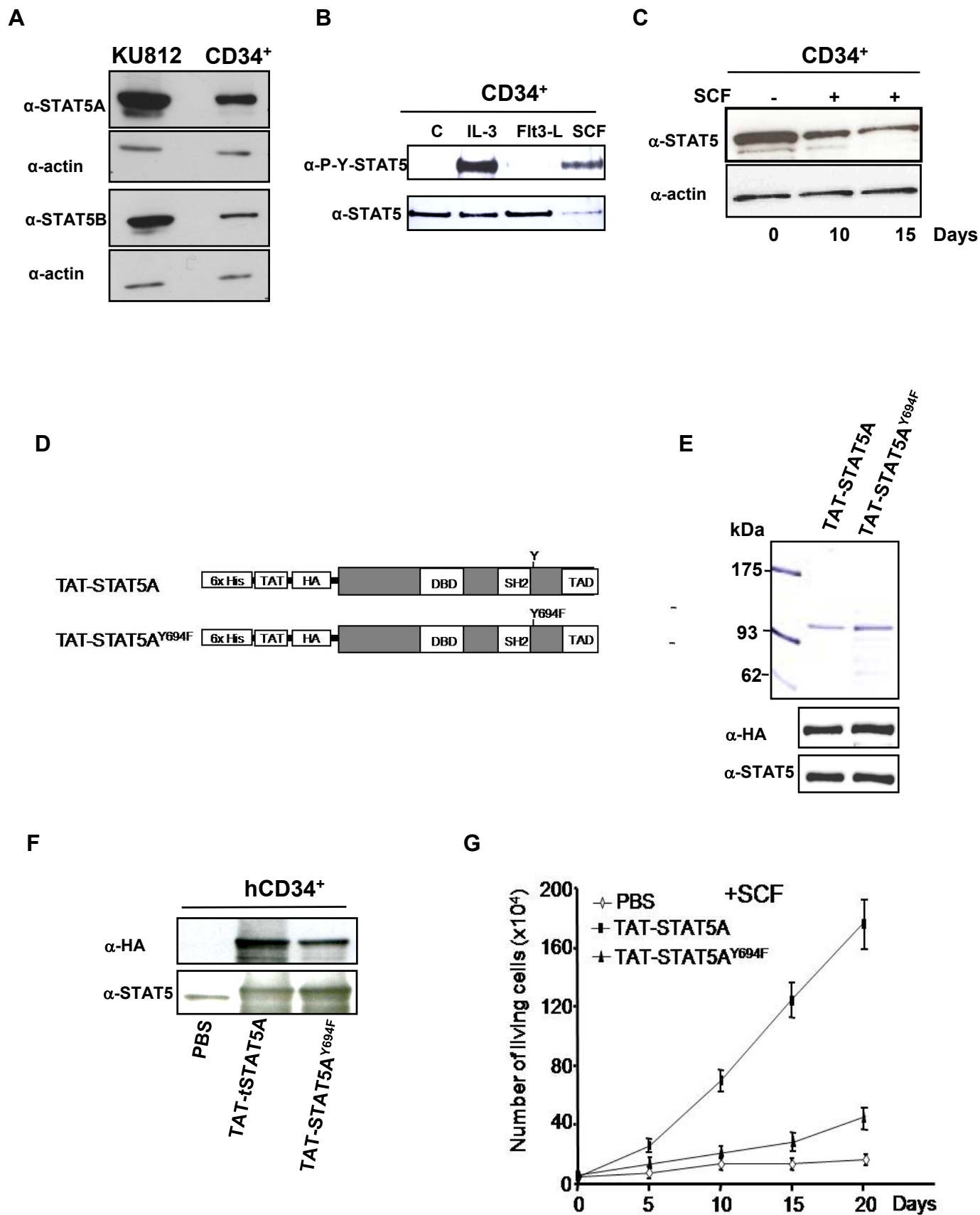

Figure S1

**A**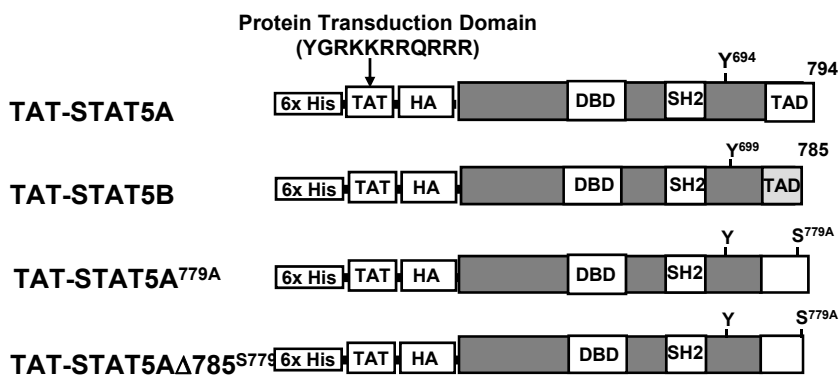**B**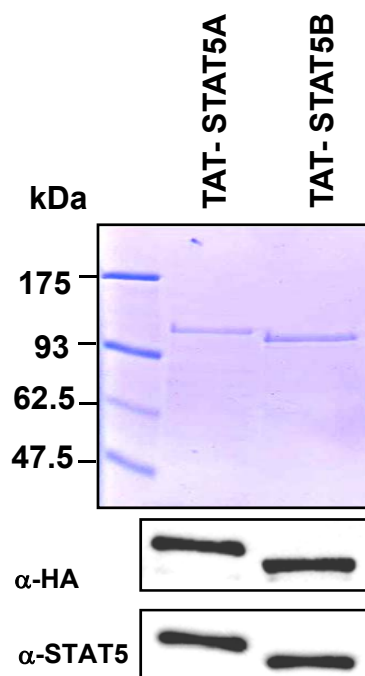**C**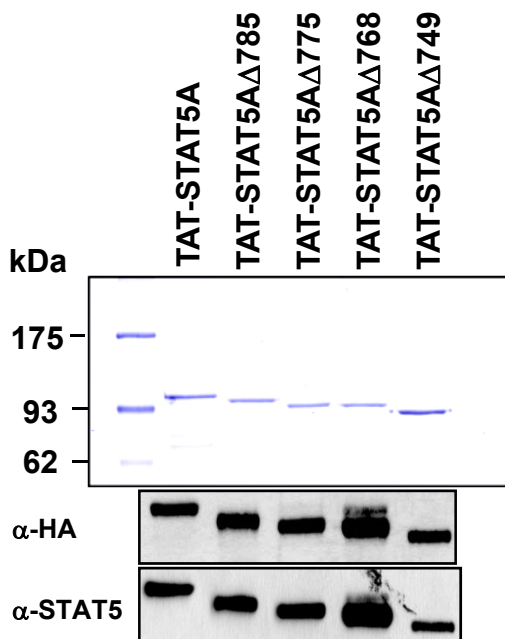**D**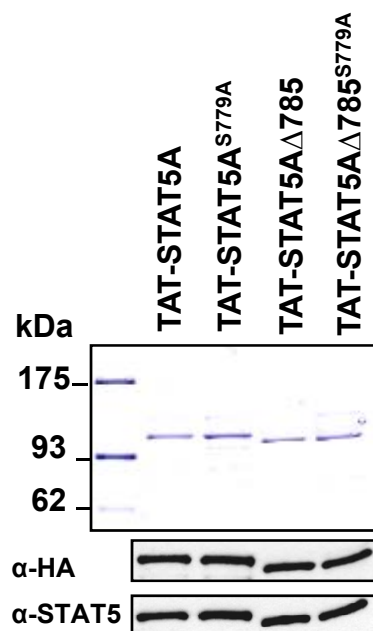**Figure S2**

**A**

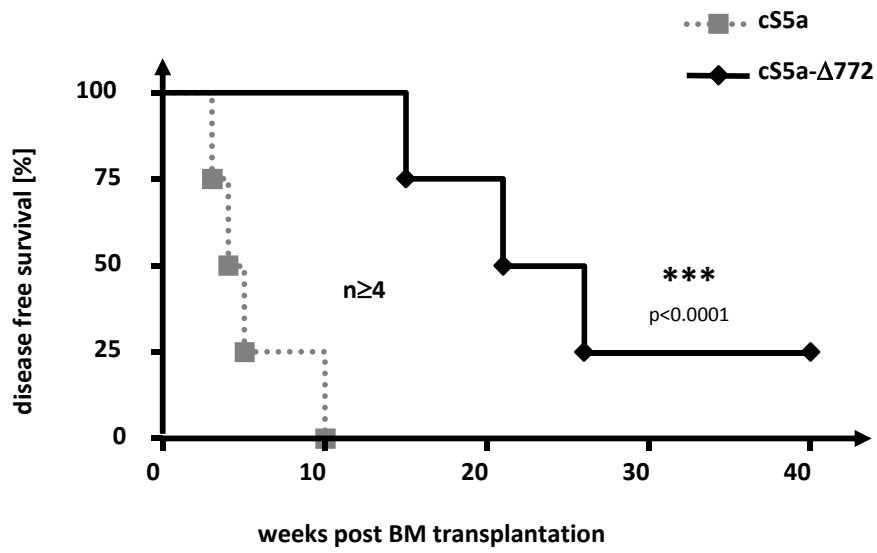

**B**

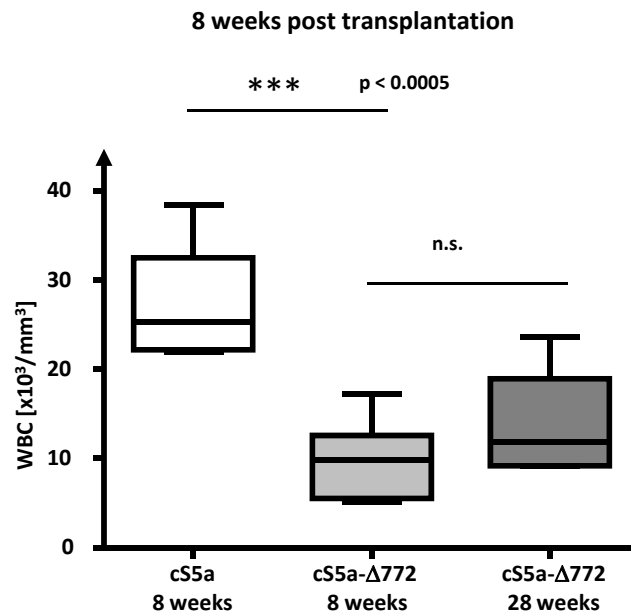

**Figure S3**

**A**

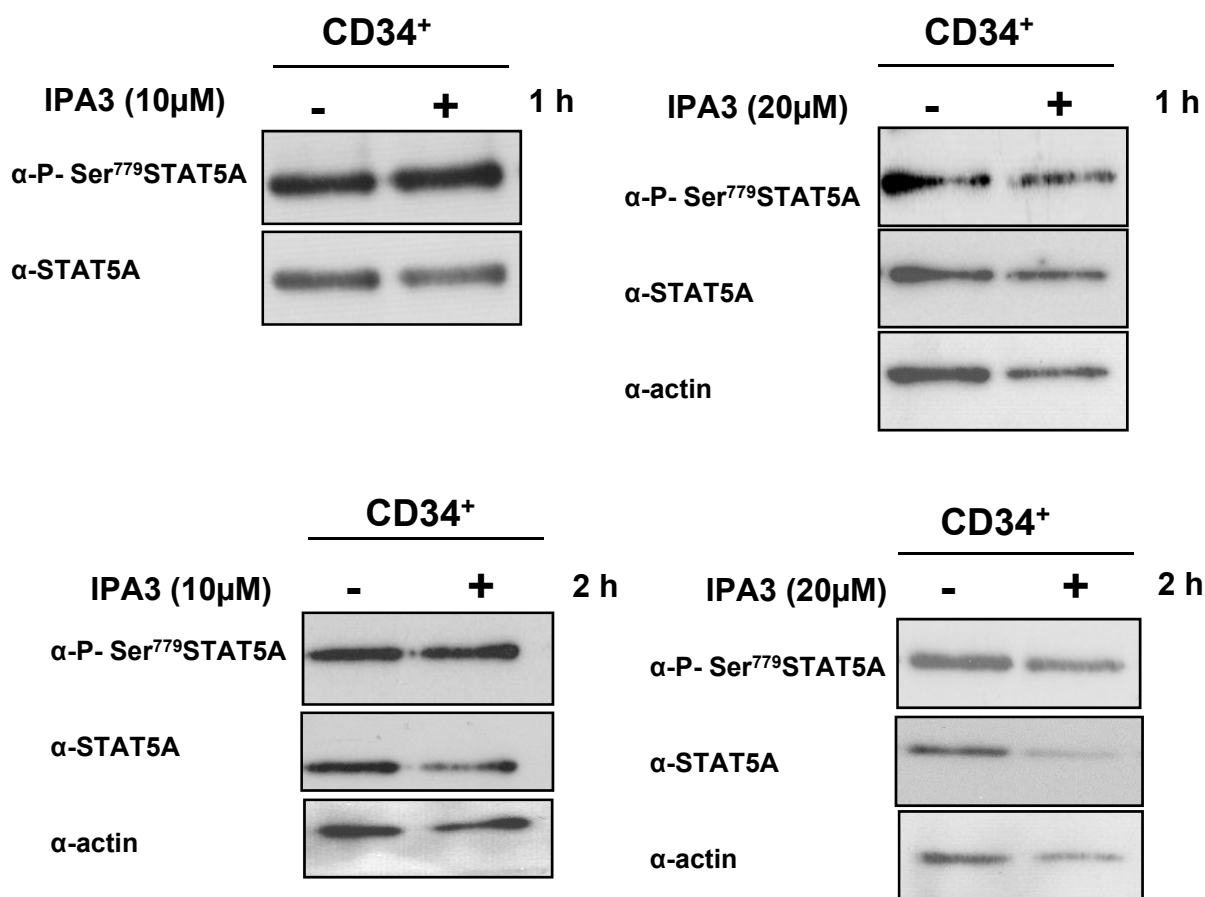

**B**

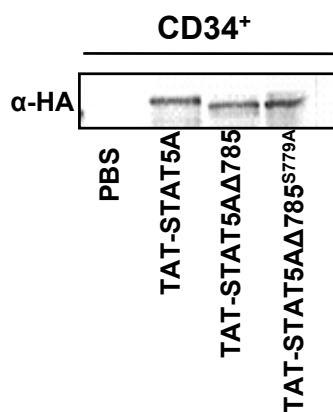

**C**

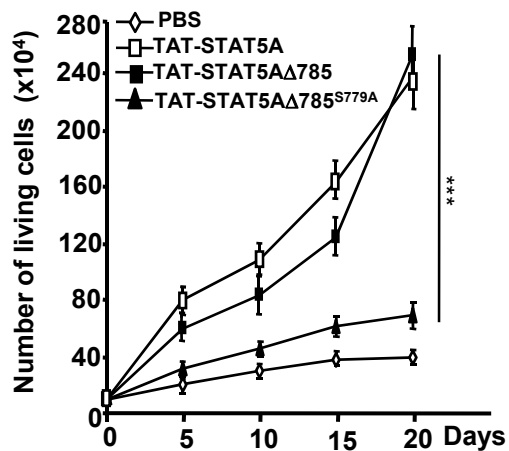

**Figure S4**

Supplement: Supplementary Figures [file bcj2016124x2.pdf]
